# Establishment and Application of Peristaltic Human Gut-Vessel Microsystem for Studying Host–Microbial Interaction

**DOI:** 10.3389/fbioe.2020.00272

**Published:** 2020-03-31

**Authors:** Bolin Jing, Zhuo A. Wang, Chen Zhang, Quanfeng Deng, Jinhua Wei, Yong Luo, Xiuli Zhang, Jianjun Li, Yuguang Du

**Affiliations:** ^1^State Key Laboratory of Biochemical Engineering, Institute of Process Engineering, Chinese Academy of Sciences, Beijing, China; ^2^Department of Chemistry, University of Chinese Academy of Sciences, Beijing, China; ^3^Key Laboratory of Fine Chemicals, Department of Chemical Engineering, Dalian University of Technology, Dalian, China; ^4^College of Pharmaceutical Sciences, Soochow University, Soochow, China

**Keywords:** peristaltic microfluidic chip, human gut-vessel co-culture model, host-microbial interaction, *Lactobacillus casei*, enteritis injury

## Abstract

Intestinal floras influence a lot of biological functions of the organism. Although animal model are strong tools for researches on the relationship between host and microbe, a physiologically relevant *in vitro* human gut model was still required. Here, a novel human gut-vessel microfluidic system was established to study the host–microbial interaction. Peristaltic motion of the cells on the chip was driven by a pneumatic pump. When intestinal epithelial cells (Caco2) were co-cultured with vascular endothelial cells (HUVECs) on the peristaltic microfluidic chip, Caco2 showed normal barrier and absorption functions after 5 days cultivation, which generally took 21 days in static Transwell models. Intestinal microvilli and glycocalyx layer were seen after 4 days cultivation, and *Lactobacillus casei* was successfully co-cultured for a week in the intestinal cavity. A model for intestinal damage and inflammatory responses caused by *E. coli* was set up on this chip, which were successfully suppressed by *Lactobacillus casei* or antibiotic. In summary, this human gut-vessel microfluidic system showed a good potential for investigating the host–microbial interaction and the effect and mechanism of microbiome on intestinal diseases *in vitro*.

## Introduction

As we all know, the intestine is an important organ capable of digestion, absorption, and secretion, establishing a protective epithelial barrier between the digestive environment and the body (Atarashi et al., 2017). Besides, the intestine is also the major site where commensal microbes of the gut microbiome live and interact with gut vascular tissues (Garrett et al., 2010) and the host immune system (Thomas et al., 2017), which contribute significantly to intestinal homeostasis (Tremaroli and Backhed, 2012). A large number of diverse microbial species reside in the distal gastrointestinal tract, and gut microbiota disorder – imbalance between the composition and function of these intestinal microbes is associated with many diseases, including inflammatory bowel disease, diabetes, obesity, cancer, and latest neurodegenerative diseases (Morgan et al., 2012; Zhao, 2013; Erny et al., 2015). In order to study the interaction between gut microbiome and host cells more simply, quickly and low-cost, there have been great efforts to develop experimental *in vitro* and *ex vivo* models of human intestine that can be used to analyze intestinal pathophysiology both in the presence and absence of living microbiome (Bartfeld, 2016; van Rijn et al., 2016; Bein et al., 2018).

Human intestinal epithelial cell lines (e.g., Caco-2 or HT-29 cells) coated by extracellular matrix (ECM) were seeded on the porous membranes within Transwell insert culture devices are one of the most commonly used *in vitro* intestinal models (Vizoso Pinto et al., 2009; Calatayud et al., 2018). Although these models are widely used for barrier function and drug absorption studies, static two-dimensional culture often requires 3 weeks for the intestinal epithelial cells to differentiate into the absorptive cells and fails to reveal some key intestinal differentiated functions (e.g., mucus production, villi formation, drug metabolism, etc.) (Kasper et al., 2016; Wang et al., 2017). Due to the overgrowth of microbes, which usually dominate the co-culture system and induce human cell apoptosis within a day, these conventional static models cannot support the coculture of commensal microbiome with human intestinal cells, which is critical for gut physiology (Rakoff-Nahoum et al., 2016).

Over the past decade, many challenges have been overcome with the development of microfluidic intestinal organ chips, which contain continuously perfused chambers to culture cells at the tissue- and organ-level in a physiologically relevant microenvironment (Vickerman et al., 2008; Bhatia and Ingber, 2014; Perrier et al., 2018). By using a computer-controlled FX5K Tension instrument and a syringe pump, Kim et al. built a physiologically relevant *in vitro* human “gut-on-a-chip,” which can achieve gut peristalsis and fluid flow, and that also has been used to study intestinal inflammation (Kim et al., 2012, 2016; Shin and Kim, 2018). The main limitation of this device is that it relies on a complex system to mechanically stretch intestinal epithelial cells rather than periodically wriggle like in the body. Shah et al. presented a modular microfluidics-based human–microbial co-culture model, HuMiX, which allows the co-culture of intestinal cells with complex microbial communities under anaerobic conditions (Shah et al., 2016). However, this study was carried out in the absence of mechanical peristalsis that can critically influence microbial growth in the intestine. Moreover, most of these studies were carried out in the absence of other supporting cells and tissue types found within the living intestine, such as endothelium-lined blood vessels and immune cells, which are important for drug transport, pharmacokinetic (PK) analysis, and disease modeling (Huh et al., 2010; Maynard et al., 2012). Furthermore, since most models formed a closed lumen through irreversible sealing, it was difficult to sample and manipulate luminal components (Pan et al., 2019; Shi et al., 2019).

In this study, we set out to develop a new human gut microsystem which aimed to overcome those limitations. Firstly, we designed a new three-channel laminated microfluidic chip that enabled multi-cell co-culture to simulate enteric cavity and vascular lumen *in vitro*. Secondly, a pneumatic pump was used to create a periodically changing pressure difference between lumen fluids of intestine and vessel, mimicking periodic peristalsis of the intestinal lumen *in vivo*. Thirdly, this laminated microfluidic device was disassembled and assembled easily with screws, so that it was convenient for cell seeding and sample collection. Cells proliferation, microvilli and glycocalyx layer secretion, barrier and absorption functions of the intestinal epithelial cells in this human gut microsystem were conveniently assessed. Finally, based on this human gut microsystem, we developed a disease model of *E. coli-*induced intestinal injury and inflammation, and assessed therapeutic effects of *L. casei* and antibiotics.

## Materials and Methods

### Fabrication and Assembly of the Microfluidic Device

The peristaltic three-dimensional human gut microsystem used in this study was fabricated from polydimethylsiloxane (PDMS, Dow Corning). The PDMS plates, which were developed by Qin et al. (2010), were fabricated by soft lithography using photoresist (SU8 3035, Microchem) as the template. All microchannel layers were individually prepared by casting PDMS prepolymer (10:1 w/w ratio of PDMS to curing agent) on a microfabricated mold of the inverse channel design made of photoresist, and curing the polymer at 60°C for 12 h. After the microchannel layers were peeled from the wafer, and peripheral holes (1.5 mm diameter) for tubing were punctured on the PDMS plates. Additionally, 5 mm diameter holes were made in the center of each PDMS plate to facilitate connections between the endothelial monolayer and the intestinal epithelial cells. The porous PDMS membranes were prepared by casting PDMS prepolymer on a microfabricated silicon wafer containing microarray with circular pillars (10 μm diameter, 30 μm height, and 25 μm spacing). The porous PDMS membranes with 5 mm diameter and 10 μm pore size were placed between PDMS plates for on-chip cell culture. After careful alignment along the vertical direction, the PDMS plates were superimposed with the top and bottom polymethyl methacrylate (PMMA) frames, and fastened with screws. Polyvinyl chloride (PVC, Watson-Marlow) tubes with an inner diameter of 25 μm were connected from three medium tanks with a multi-channel pneumatic pump (MFCS-EZ, FUIGENT) to the upper medium and lower microfluidic channels, respectively. This allowed us to regulate application of air pressure to the fluid medium by computer to exert cyclic change of fluid pressure within each of the microchannel to mimic peristaltic motions.

### Cells Culture

The human intestinal epithelial cells Caco2 from ATCC were cultured in Dulbecco’s Modified Eagle Medium containing 4.5 g^∗^L^–1^ glucose medium (DMEM/HG, Gibco) supplemented with 10% (w/v) of fetal bovine serum (FBS, Gibco). The human umbilical vein endothelial cells (HUVECs) from ATCC were cultured in DMEM/F12 medium (DMEM/F12, Gibco) containing 10% (w/v) of fetal bovine serum. Human macrophage U937 cells from ATCC were cultured in RPMI 1640 medium (RPMI 1640, Gibco) containing 10% (w/v) of FBS. Penicillin (100 units/mL, Gibco) and streptomycin (100 μg/mL, Gibco) were added to all aforementioned media. All cells were cultured in a cell incubator with 5% CO_2_ at 37°C. Antibiotics were removed from the culture medium for co-culture of human cells with living intestinal microbes.

### Caco2 Cells Culture on the Transwell

For static intestinal epithelial absorption model, 1 × 10^5^ cells/cm^2^ Caco2 cells stained by CellTrackerTM Green CMPTX (5 mM) were plated in the top chamber with collagen type I hydrogel coated membrane (6.5 mm diameter, 10 μm pore size). Then culture medium was added into the upper chamber and the lower chamber.

### Establishing the Coculture Model of Caco2 and HUVECs on the Chip

Before the microdevice was assembled, all tubes and PDMS plates were pre-treated with 75% ethanol for 12 h, and the entire system was then dried in oven at 60°C. The dried devices were exposed to ultraviolet light for 30 min. The porous membrane was coated with collagen type I hydrogel at 37°C. The Caco-2 cells (1 × 10^5^ cells/cm^2^) stained by blue cell-tracker (CMAC Dye, Invitrogen) were seeded on the porous PDMS membrane and incubated at 37°C for 3–4 h, allowing the seeded intestinal epithelial cells to attach to the membrane surface. Then, HUVECs (1 × 10^5^ cells/cm^2^), which were stained by green cell-tracker (CMFDA Dye, Invitrogen) and wrapped by type I collagen gel, were seeded onto the basal of the membrane. After vascular endothelial cell attached to the membrane, the microdevice was assembled and ran as described above.

### Establishing the Inflammatory Bowel Disease Model Caused by *E. coli* on the Chip

The Escherichia coli strain (11775, ATCC) was purchased from China General Microbiological Culture Collection Center (CGMCC). It was cultivated in autoclaved LB medium at 37°C and 200 rpm for 12 h. After bacterial cell density was adjusted to ∼1.0 × 10^7^ CFU/mL, *E. coli* was spun down (10,000 g, 8 min), and resuspended in antibiotics-free DMEM medium with 5 μmol/mL red cell-tracker (CMTPX Dye, Invitrogen), and cultivated at 37°C for 30 min. Then E. coli was spun down, and resuspended in antibiotics-free DMEM + 10 FBS medium immediately and flowed into the intermediate microchannel containing the villus epithelium pre-cultivated in the microfluidic chip with peristalsis plus vascular endothelial cell for 4 days. At the same time, macrophages (4 × 10^5^ cells/mL) were introduced into the vascular lumen. After E. coli cells and macrophages cells were allowed to attach to the surface of epithelium or endothelium during ∼2 h under static condition, fresh antibiotics-free culture medium was perfused into all three microchannels at 60 μL/h with cyclic peristalsis (15%, 0.15 Hz).

### Anti-inflammatory Evaluation of *L. casei* and Antibiotics on the Chip

*Lactobacillus casei* L5 BGB (*L. casei* L5 BGB) was isolated from the human intestine by our group, which was basically similar to *Lactobacillus casei* from ATCC based on the phylogenetic tree (Supplementary Figure S3). For the pre-cultivation of *L. casei* L5 BGB, colonies on solid medium were resuspended in the mixture of sterilized Lactobacilli MRS Broth (Difco, BD Diagnostics) and cultivated at 37*°*C at 200 rpm for 12 h. After cell density was adjusted to ∼1.0 *×* 10^7^ CFU/mL, *L. casei* L5 BGB was mixed with *E. coli* and introduced into the intermediate epithelium-lined channel of the gut-vessel microsystem as described above. About anti-inflammatory experiments of antibiotics, penicillin (100 units/mL, Gibco) and streptomycin (100 μg/mL, Gibco) were added to the culture medium of the intermediate chamber after *E. coli* cells attached to the surface of epithelium.

### Analysis of Cell Viability on the Chip

The live/dead cell imaging kit (R37601, Life Technologies) was used to evaluate cell viability on the microfluidic device. In the live/dead cell assay, the viable cells were stained green, while the dead ones were stained red. The cells were loaded onto the microfluidic device. After several days of cultivation, the culture chambers on the microfluidic chip were flushed with phosphate buffered saline (PBS, HyClone) for 1–3 min. The cells were then incubated with the live/dead cell imaging reagents for 15–30 min at 37°C. Next, the culture chambers were flushed with PBS for 3–5 min to remove the reagents and observed under a fluorescent microscope. The percentage of cell viability was calculated by dividing the number of viable cells (green) by the total number of cells.

### Morphological Studies

Morphological observation was done by following the standard protocol. After cultivation, the Caco2 cells were fixed with 4% paraformaldehyde (28908, Thermo Fisher Scientific) for 15 min, and permeabilized with 0.1% Triton X-100 (HFH10, Thermo Fisher Scientific) in PBS for 10 min, and blocked with 3% BSA (30036727, Thermo Fisher Scientific) in PBS for 30 min at room temperature. To visualize epithelial tight junctions, cells were then incubated with occludin monoclonal antibody (331588, Thermo Fisher Scientific) at 10 μg/mL in blocking buffer for 1 hr at room temperature, and washed with PBS. To visualize the protein of intestinal epithelial mucus layer, cells were then incubated with villin polyclonal antibody (PA5-29078, Invitrogen) at 10 μg/mL in blocking buffer for 1 h at room temperature, and washed with PBS, and incubated with a Goat anti-Rabbit IgG (H + L) Super clonal Secondary Antibody (A10474, Life Technologies) and Alexa Fluor^®^ 594 conjugate at a dilution of 1:1000 for 1 hr at room temperature. To visualize the carbohydrate of intestinal epithelial mucus layer, cells were then incubated with fluorescein isothiocyanate labeled Wheat germ agglutinin (WGA, Sigma) for 30-45 min at 37°C, then washed with PBS. All of the nuclei were stained blue with DAPI (D1306, Invitrogen). Images were taken under an inverted laser scanning confocal microscope.

### Measurement of Paracellular Permeability

The barrier-forming capacity of the intestinal epithelial monolayer formed by Caco2 cells and vascular endothelial monolayer formed by HUVECs were evaluated by measuring the apparent permeability (*P*_app_) of FITC-labeled dextrans with different molecular weights (10, 40, and 70 kDa, Sigma) through the monolayer. One milliliter of D’Hanks solution containing FITC-dextrans (2 nmol/mL) with different molecular weights was perfused through the microchannel of the middle PDMS layer, and the blank D’Hanks solution was circulated through the upper and lower microchannel at a flow rate of 60 μL/h. *P*_app_ was calculated using the equation below (Gao et al., 2013):

(1) Papp[cm/s]=(1/A*C0)(dQ/dt)

where *A* = area of mass transfer, *C*_0_ = donor concentration of reagent in the upper medium, and *dQ/dt* = transmembrane transportation rate.

### Measurement of Aminopeptidase Activity

The function of human intestinal epithelial cells was evaluated by measuring the specific activity of an apical brush border aminopeptidase enzyme that is expressed by differentiated human intestinal Caco2 cell using L-alanine-4-nitroanilide hydrochloride (A4N, Sigma) as a substrate (Piana et al., 2007). To measure the specific activity of aminopeptidases in the microfluidic device, the A4N solution was flowed through the middle microchannel of the device containing two Caco2 layers cultured in the presence or absence of endothelial cells. Samples (100 μL) were collected every 2 h from the outlet of the middle microchannel and transferred to a 96 well plate (Black/clear flat bottom, BD Falcon) where the cleavage product (i.e., 4-nitroaniline) was quantified in a microplate reader (SpectraMax M5, Molecular Devices) at 405 nm using culture medium as a reference. The activity of aminopeptidase enzyme expressed by Caco2 cell monolayers cultured under static conditions in the Transwell was also measured.

### Inflammatory Responses

To analyze inflammatory cytokines, culture broth was collected from the outlet of both intestinal cavity and vascular lumen, then immediately frozen at -80°C until the analysis was performed. Amounts of cytokines (IL-8, IL-6, TNF-α, and IL-1β) in each sample (*n* = 3) were analyzed using an ELISA kit (KHC0082, KHC0062, KHC0012, KHC3012; Life Technologies) according to the manufacturer’s protocol.

### Data Analysis and Quantification

All data were analyzed by averaging the values of at least three microfluidic devices, with each device representing one independent experiment. All results and error bars in this article are represented as mean ± SE (SEM). Student’s tests (*n* = 3, *P* < 0.05) and correlation analysis were performed with GraphPad Software.

## Results

### Microfabrication of the Peristaltic Human Gut-Vessel Microsystem

**FIGURE 1:**
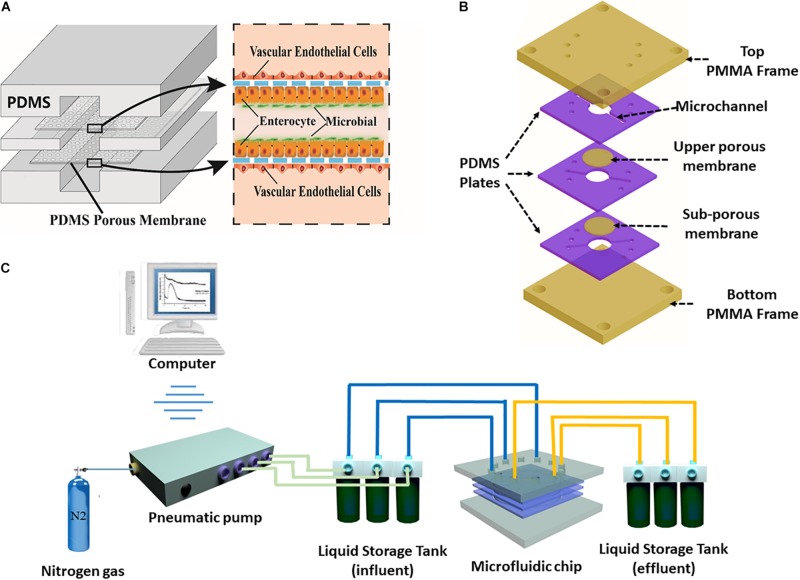
The peristaltic human gut-vessel microsystem. **(A)** Conceptual diagram of the microsystem of human gut representing co-culture of vascular endothelial cells, intestinal epithelial cells with intestinal microbes. **(B)** Annotated exploded view of the microfluidic device used in this study. **(C)** Diagram of the experimental set-up of the peristaltic human gut-vessel model with pneumatic pump system for the perfusion of culture media.

The human gut-vessel microsystem was designed to simulate the periodic peristalsis of the intestinal cavity, and study drug absorption into the blood and interaction between the intestinal microbes and the host. To achieve these goals, the laminated microfluidic device consists of three layers of PDMS plates with length of 30 mm and thickness of 2 mm. The PDMS plates sandwiched two layers of porous PDMS membranes coated with ECM. Two pieces of PMMA plates as frames were fixed with screws to stabilize the whole device, as shown in Figure 1B and Supplementary Figures S1A–C. Microchannels with width of 1 mm and height of 100 μm were fabricated on each layer of PDMS plate. Caco-2 cells were seeded on both the upper membrane and the lower membrane layer in the middle microchannel, whereas HUVECs were seeded on the other side of the two membranes in the upper and lower microchannels, as shown in Figure 1A and Supplementary Figure S1D. By using a computer-controlled pneumatic pump (Figure 1C), culture medium was perfused at desired flow rates through each microchannel, mimicing the fluid flow and associated shear stress on the cell surface in the human intestinal lumen and the blood vessels *in vivo*.

### Peristaltic Motion on the Microsystem

**FIGURE 2:**
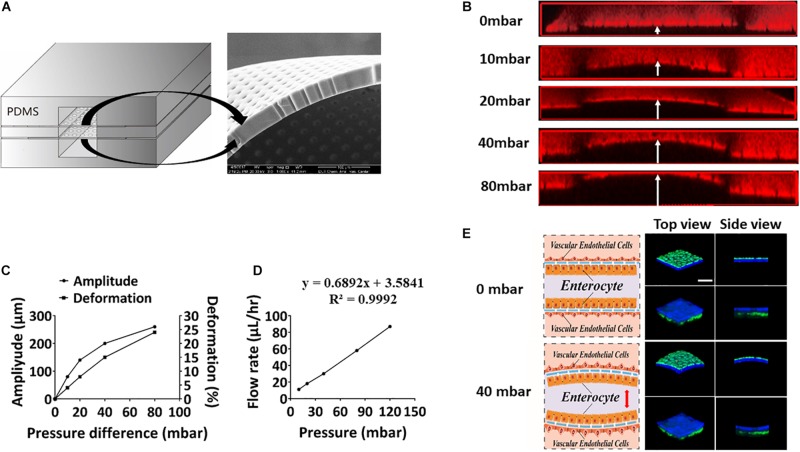
Evaluation of peristaltic effect in the microsystem. **(A)** A cross-sectional view of the porous membrane on the microfluidic chip (left), and scanning electron micrograph showing a side view of a portion of the porous membrane (right). **(B)** Laser confocal layer scanning diagram of the deformation of the PDMS porous membrane on the microfluidic device under different fluid pressure differences. Red rhodamine solution was flowed through the middle layer of the chip, and colorless deionized water was flowed through the upper and lower layers. **(C)** Quantitation of the amplitude produced in the ECM-coated, flexible, porous PDMS membrane and deformation produced in the adherent intestinal epithelial cells under different fluid pressure differences. **(D)** Quantitative relationship between the fluid flow rate and the air pressure on the microsystem. **(E)** Schematics (left) and laser confocal layer scanning diagrams (right) of the deformation of the intestinal epithelial layer (blue) and vascular endothelium (green) adhering to the porous membrane cultured within the gut-vessel microfluidic chip at fluid pressure differences of 0 and 40 mbar (bar, 50 μm).

To mimic periodic peristalsis of the intestinal cavity in the microfluidic device, culture medium was flowed through the upper and lower microchannels at a constant pressure. Culture medium in the middle microchannel was driven by the pneumatic pump with cyclic pressure variations. Thus, pressure difference periodically existed between the middle microchannel and the upper/lower microchannel, driving periodic peristalsis of the intestinal epithelial cells adhering to the porous PDMS membrane in the middle microchannel. The size of the pores and the flexibility of the porous PDMS membrane were clearly observed under scanning electron microscopy (Figure 2A), and the amplitude and deformation of the porous PDMS membrane were measured under different fluid pressure differentials. As shown in Figures 2B,C, when the fluid pressure difference was 40 mbar, the deformation of the membrane reached about 15%, which was very similar to the mechanical microenvironment of epithelial cells *in vivo* (Martinez-de-Juan et al., 2000). To create fluid shear stress on the cells surface close to that *in vivo*, the relationship between flow rate and fluid pressure in the microchannel was assessed. As shown in Figure 2D, the flow rates increased linearly from 0 to 85 μL/h as the fluid pressures were raised from 0 to 120 mbar. And when the fluid pressure was 80 mbar, the flow rate reached 60 μL/h, which produced 0.04 dyne/cm^2^ shear stress similar to that under physiological conditions (Gray and Stroka, 2017). Thus, to simulate physiological conditions (Kim et al., 2012), the fluid pressure in the vascular lumen was constant at 80 mbar (60 μL/h, 0.04 dyne/cm^2^) and that in intestinal lumen was varied from 40 mbar to 120 mbar periodically (0. 15 Hz). Caco2 cells were labeled with cell-tracker blue and HUVECs were labeled with cell-tracker green, it was shown under a laser scanning confocal microscope that both intestinal epithelial layer and vascular endothelium underwent significant deformation at a pressure difference of 40 mbar (Figure 2E).

### Impact of Peristalsis and Endothelial Cells on Growth of Epithelial Organization

**FIGURE 3:**
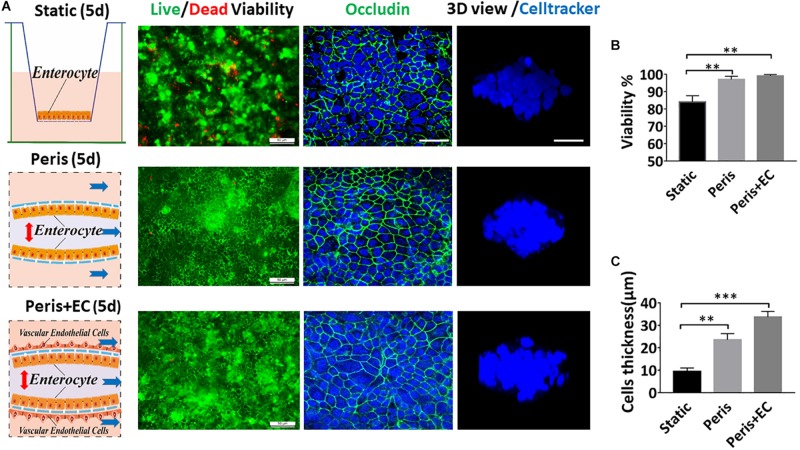
Impact of peristalsis and endothelial cells on epithelial organization growth. **(A)** Growth of the Caco-2 epithelial cells cultured in the static Transwell system (Static) versus in the peristaltic microfluidic chip without (Peris) or with endothelial cells (HUVECs; Peris + EC) for 5 days. Schematics (left) showing the system layout; the confocal fluorescence views (center) showing the viability of the Caco2 cells (live cells in green, dead in red) and the distribution of the tight junction protein – occludin in the epithelial monolayers (nuclei in blue, occludin in green); and the laser confocal scanning views (right) showing a vertical cross section of the epithelium highlighting cell shape (bar, 50 μm). Statistical analysis of the viability **(B)** and the height **(C)** of intestinal epithelial cell cultured in the static Transwell system or in the peristaltic microfluidic chip without or with endothelial cell for 5 days (*n* = 3; **p < 0.01, ***p < 0.05).

To explore the physiological relevance of mimicking the physical microenvironment of the intestine, Caco-2 cells were grown either in a static Transwell chamber (Figure 3A) or in the microfluidic device with peristalsis (Figure 3B). After 5 days of cultivation, 97 (± 1.8)% Caco-2 cells remained alive in the micro-device with peristalsis, which was much higher than 84 (± 3.6)% in the Transwells (Figure 3D). Immunostaining data showed the bright signals of the tight junction protein – occludin at the edge of cells, suggesting that Caco-2 cells formed confluent polygonal epithelial monolayers with well-developed tight junctions in the microfluidic device, much tighter than cells in the Transwell (Figures 3A–C). The thickness of cell layers grown in the microfluidic device with peristalsis reached 23.6 (± 2.8) μm, whereas that was only 9.4 (± 1.5) μm in Transwell (Figure 3E). Moreover, Caco-2 cells co-cultured with HUVEVs in the microfluidic device with peristalsis also showed 99 (± 1.8)% cell viability (Figure 3D) and even thicker cell layers [33.6 (± 2.5) μm] (Figure 3E).

### Reconstitution of Intestinal Physical Barrier Function and Absorption in the Microfluidic Device

**FIGURE 4:**
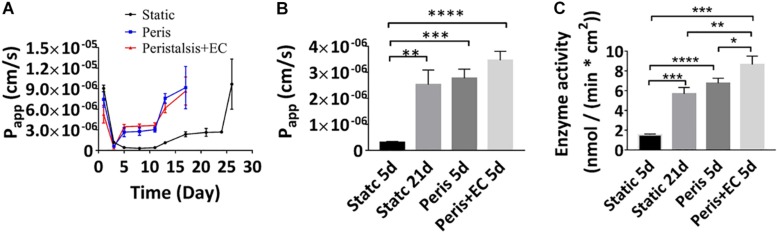
Reconstitution of intestinal barrier and absorbent functions *in vitro*. **(A)** Apparent paracellular permeability (*P*_app_) measured by real-time quantitating fluorescent dextran transport through the Caco-2 monolayer cultured under static conditions for 26 days, or in the peristaltic microfluidic chip without or with endothelial cell for 17 days. Tight junctional integrity and the differentiation of the epithelium assessed by measuring the *P*_app_ values **(B)** and the brush border aminopeptidase activity **(C)** of the intestinal epithelial cells cultured in the static Transwell system for 5 days or 21 days, or in the peristaltic microfluidic chip in the absence (Peris) or presence (Peris + EC) of endothelial cell for 5 days (*n* = 3; *p < 0.001, **p < 0.01, ***p < 0.05, ****P < 0.0001).

The Transwell model of intestinal epithelial barrier function that is often used as a tool for drug screening as well as cell biological studies, involves culture of Caco-2 cells on a porous Transwell membrane for 21days, tight junctional integrity and absorption function are generally measured by quantifying the *P*_app_ value of the intestinal epithelium. We therefore compared *P*_app_ value of Caco-2 layers grown under static Transwell conditions with those in the microfluidic device with peristalsis alone and peristalsis plus endothelial cells using fluorescent-labeled 10 kDa dextran (FD10k). Real time monitoring showed that the *P*_app_ value of Caco2 cells cultured in a static Transwell chamber reached a minimum of 3.1 (± 0.32) ^∗^10^7^ cm/s on the 8th day, and Caco2 cells cultured for 3 days with or without HUVECs in the microfluidic device with peristalsis exhibited a similar Papp value (Figure 4A). Besides, a stable *P*_app_ value of Caco2 layer [2.4 (± 0.37) ^∗^10^–6^ cm/s] cultured in a static Transwell chamber was reached on the 19th day (Figure 4A), and it only took 5 days to achieve similar *Papp* in the microfluidic device with or without HUVECs. By contrast, the stable *P*_app_ value of Caco2 cells cultured in the microfluidic device with peristalsis plus HUVECs was higher than that in the microfluidic device without HUVECs after 5 days of cultivation (Figure 4B). The *P*_app_ value of FD10k through the vascular endothelium monolayers during 7 days were also measured (Supplementary Figure S2A), it reached a stable value 4.1 (+ 1.0) ^∗^10^–6^ cm/s on the 3rd day. Noticeably, the *P*_app_ values of HUVECs monolayer on day 5 [4.1 (± 1.0) ^∗^10^–6^ cm/s] was significantly higher than that of the Caco2 monolayers (Supplementary Figures S2B,C).

The catalytic activity of aminopeptidase represents cytodifferentiation of Caco-2 cells. Our data showed that, after 21 days of cultivation in the static Transwell system, the aminopeptidase activity [5.69 (± 0.63) nmol/(min ^∗^ cm^2^)] in Caco-2 cells was fivefold higher than that of 5-day of culture [1.23 (± 0.08) nmol/(min ^∗^ cm^2^)] (Figure 4C), indicating an increase in cell differentiation, consistent with previously published findings (Howell et al., 1992). Importantly, peristalsis greatly accelerated cell differentiation in the microfluidic device, resulting in almost an eightfold increase in aminopeptidase activity in Caco-2 cells after 5-day of co-culture with HUVECs [8.65 (± 0.85) nmol/(min ^∗^ cm^2^)] (Figure 4C).

### Evaluation of Intestinal Epithelial Glycocalyx and Microvilli

**FIGURE 5:**
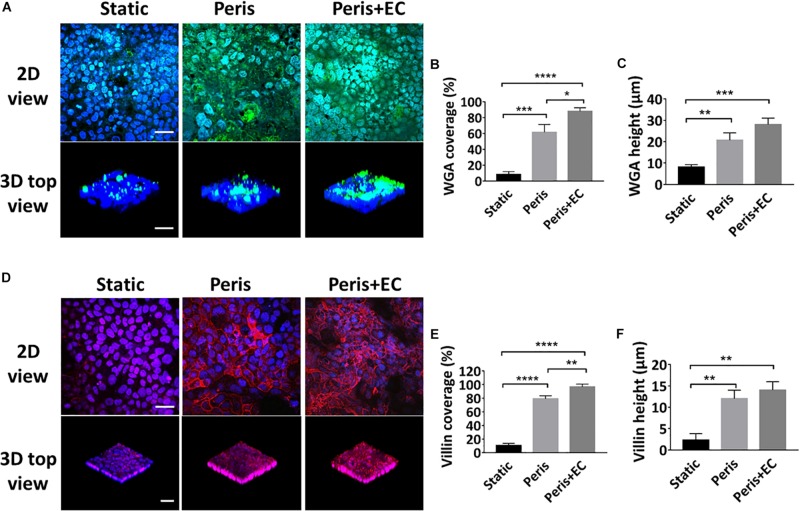
Evaluation of intestinal epithelial glycocalyx and microvilli. **(A)** Confocal immunefluorescence views of the sugar chain portion (FITC-WGA, green) of the glycocalyx layer secreted by the Caco2 cells cultured in the static Transwell system, or in the peristaltic microfluidic chip in the absence (Peris) or presence (Peris + EC) of endothelial cell for 5 days (bar, 50 μm). Computerized quantification of the coverage **(B)** and height **(C)** of the sugar chain portion marked by FITC-labeled WGA measured under the conditions described in **(A)** (*n* = 3; *P < 0.05, **p < 0.01, ***P < 0.001, ****P < 0.0001). **(D)** Confocal immunofluorescence views of the villin (red) of the microvilli secreted by the Caco2 cells cultured in the static Transwell system, or in the peristaltic microfluidic chip in the absence (Peris) or presence (Peris + EC) of endothelial cell for 5 days (bar, 50 μm). Quantification of the coverage **(E)** and height **(F)** of villin done under the conditions as described in **(D)** (*n* = 3; **p < 0.01, ****P < 0.0001).

Glycocalyx and microvilli are the efficient defense system for protecting the epithelium from pathogens by promoting their clearance and separating them from the epithelial cells (Liu et al., 2017). To investigate the secretion of glycocalyx and microvilli by intestinal epithelial cells in the microsystem, the expression levels of sugar chain and villin were evaluated (Figures 5A,D). Statistical analysis showed that the 61 (± 10.0)% coverage and 21 (± 3.5) μm height of the glycan of the glycocalyx layer was secreted by Caco-2 cells cultured in the peristaltic microfluidic chip, much higher than 5 (± 2.2)%) coverage and 8 (± 1.5) μm height of the glycocalyx glycan in the static Transwell chamber (Figures 5B,C). Besides, Caco2 cells cultured in the peristaltic microfluidic chip showed the 79 (± 4.6)% coverage and 12 (± 2.0) μm height of the villin, while those under static conditions were only 10 (± 3.1)% and 2 (± 1.5)% μm, respectively (Figures 5E,F). Surprisingly, the coverage of glycan (88 ± 4.6%) and villin (97 ± 4.2%) were significantly increased when Caco-2 cells were co-cultured with HUVECs in the peristaltic microfluidic chip, though the height of the glycan and villin were not obviously affected (Figure 5).

### Host Cells and *L. casei* Co-culture on the Peristaltic Human Gut-Vessel Microsystem

**FIGURE 6:**
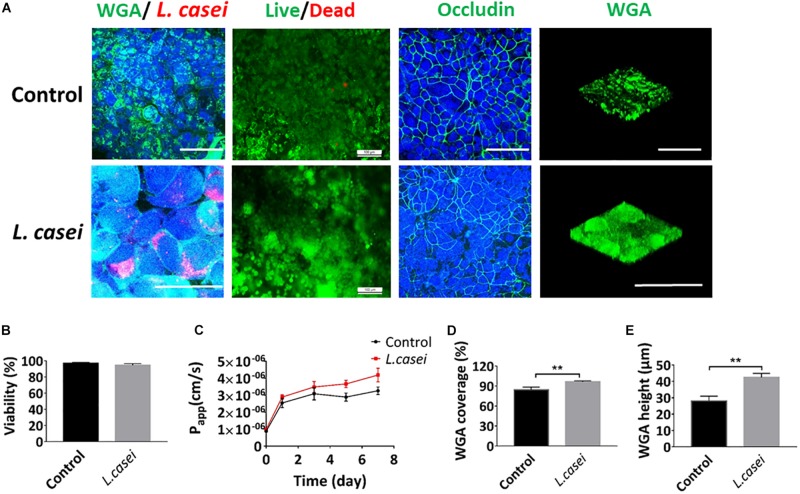
Co-culture of Caco2 and *L. casei* in the peristaltic human gut-vessel microsystem. **(A)** Confocal immunofluorescence views of the colonization of probiotics (*L. casei L5 BGB* in red, WGA in green), the viability of the Caco2 cells (live cells in green, dead cells in red), the distribution of the tight junction protein – occludin in the epithelial monolayers (nuclei in blue, occludin in green) and sugar chain portion (FITC-WGA, green) of the glycocalyx layer secreted by the Caco2 cells cultured in the peristaltic human gut-vessel microsystem with (bottom) or without (top) probiotics (*L. casei L5 BGB*, red) for 7 days (bar, 100 μm). **(B)** Statistical analysis of the viability of intestinal epithelial cell cultured under the conditions described in **(D)** (*n* = 3). **(C)** Changes in *P*_app_ measured by quantitating fluorescent dextran transport across the tissue-tissue interface within the peristaltic human gut-vessel microsystem with (*L. casei*) or without (Control) *L. casei L5 BGB* for 7 days. Quantification of the coverage **(D)** and height **(E)** of the sugar chain portion marked by FITC-labeled WGA done under the conditions as described in **(A)** (*n* = 3; **p < 0.01).

The presence of microbial communities in the lumen of the gut **in vitro** is one of the most crucial components of human gut physiology that have never been modeled effectively on static Transwell chamber (O’Hara and Shanahan, 2006). To assess co-culture of microbial flora and human cells in the peristaltic human gut-vessel microsystem, the commensal intestinal microbe – **L. casei** L5 BGB was cultured in the intestinal cavity. When the culture solution DMEM + 10% FBS was flowed through the intestinal lumen, both pH and the *P*_app_ value of intestinal cavity were maintained at a relatively stable state during 7 days of co-culture (Supplementary Figures S4A,B). After 7 days of co-cultivation with *L. casei*, coverage and height of the glycocalyx layer increased significantly, reaching 100 ± 3.0% and 43 ± 2.4 μm respectively (Figures 6A,D,E). Colonization of *L. casei* on the surface of the Caco2 cells was also confirmed. Moreover, the pH value in the intestinal lumen of the co-culture group (pH 6.3) was lower than the control group (pH 7.0) (Supplementary Figure S4B). The viabilities of both Caco2 cells and *L. casei* L5 BGB maintained above 97% after 7 days of co-culture (Figure 6B). Furthermore, occludin expression of Caco2 cells remained intact, and the *P*_app_ value of intestinal epithelium was 3.8 (± 0.3)^∗^10^–6^ cm/s after 7 days co-culture in the peristaltic microsystem, slightly higher than that of the control group (Figure 6C). Thus, the barrier function of intestinal epithelial cells remain intact after co-culture with *L. casei*.

### Intestinal Damage and Inflammatory Responses Caused by *E. coli* on the Chip

**FIGURE 7:**
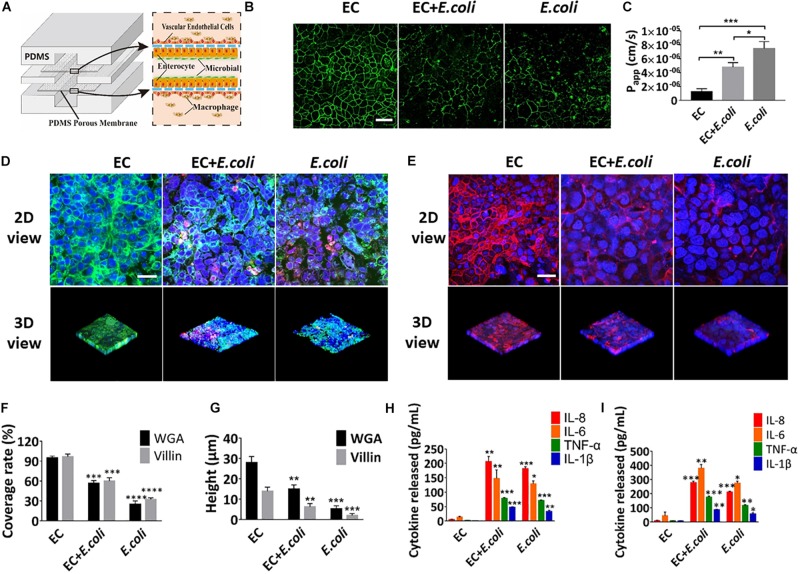
Intestinal damage and inflammatory responses caused by *E. coli* on the chip. **(A)** Conceptual diagram of the microsystem of human inflammatory bowel disease representing co-culture of vascular endothelial cells, enterocyte, macrophages with enterobacteria. **(B)** The confocal fluorescence views showing the distribution of the tight junction protein – occludin (green) in the epithelial monolayers cultured in the peristaltic human gut-vessel microsystem under control conditions (EC) versus presence of *E. coli* with (EC + *E. coli*) or without (*E. coli*) endothelial cells for 24 h (bar, 50 μm). **(C)** Quantification of *Papp* measured by quantitating fluorescent dextran transport across intestinal epithelial layer under the conditions as described in **(B)** (*n* = 3; *P < 0.05, **p < 0.01, ***P < 0.001). **(D)** Confocal immunofluorescence views of the sugar chain portion (FITC-WGA, green) of the glycocalyx layer secreted by the Caco2 cells cultured under the conditions described in **(B)** (nuclei in blue, *E. coli* in red) (bar, 50 μm). **(E)** Confocal immunofluorescence views of the villin of the microvilli secreted by the Caco2 cells cultured under the conditions described in **(B)** (nuclei in blue, villin in red) (bar, 50 μm). Computerized quantification of the coverage **(F)** and height **(G)** of the sugar chain portion and villin measured under the conditions described in **(B)** (*n* = 3; **p < 0.01, ***P < 0.001, ****P < 0.0001). Polarized secretion of proinflammatory cytokines (IL-8, IL-6, TNF-α, and IL-1β) in the enteric cavity **(H)** and vessel lumen **(I)** under the conditions described in **(B)** (*n* = 3; *P < 0.05, **p < 0.01, ***P < 0.001).

Some key hallmarks of inflammatory intestinal diseases are destruction of intestinal epithelia and secretion of inflammatory cytokines, which are believed to result from complex pathological interplay among the intestinal epithelium, gut microbes, and immune cells, as well as changes in luminal flow caused by altered peristalsis (Fiocchi, 1998). To explore whether this microsystem could be used to model human intestinal inflammation *in vitro*, *E. coli* was inoculated into the intestinal lumen and human macrophages U937 cells were introduced into the vascular cavity with or without vascular endothelial cells (Figure 7A). Addition of *E. coli* significantly affected the coverage and height of the glycocalyx and microvilli after 24 h of co-cultivation on the chip (Figures 7D–G) (quantified). Change on the expression level of intercellular tight junction protein and the *P*_app_ value of the intestinal epithelium, indicated that intestinal epithelial barrier function was also greatly destroyed within 24 h (Figures 7B,C). Besides, when *E. coli* was colonized in the intestine cavity, there was a significant increase (*P* < 0.01) in secretion of NO, TNF-α, IL-1β, IL-6, and IL-8 into both enteric cavity and vascular lumen (Supplementary Figures S5A,B and Figures 7H,I). And the inflammatory factors secreted by the vascular chamber were significantly higher than the intestinal lumen. Unexpectedly, damage of glycocalyx, microvilli and barrier function were aggravated in the absence of vascular endothelial cells, whereas the secretion of inflammatory factors was reduced in all chambers (Figure 7).

### Anti-inflammatory Evaluation of *L. casei* and Antibiotics in the Microsystem

**FIGURE 8:**
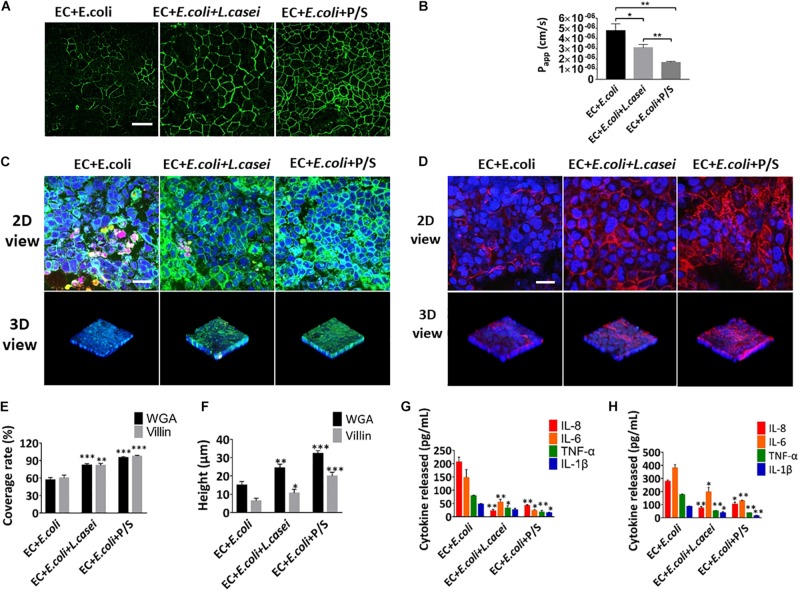
Anti-inflammatory evaluation of *L. casei* and antibiotics on the chip. **(A)** The confocal fluorescence views showing the distribution of the tight junction protein – occludin (green) in the epithelial monolayers cultured in the peristaltic human gut-vessel microsystem with *E. coli* under control conditions (EC + *E. coli*) versus presence of *L. casei* (EC + *E. coli* + *L. casei*) or P/S (EC + *E. coli* + P/S) for 24 h (bar, 50 μm). **(B)** Quantification of *Papp* measured by quantitating fluorescent dextran transport across intestinal epithelial layer under the conditions described in **(A)** (*n* = 3; *P < 0.05, **p < 0.01). **(C)** Confocal immunofluorescence views of the sugar chain portion (FITC-WGA, green) of the glycocalyx layer secreted by the Caco2 cells cultured under the conditions described in **(A)** (nuclei in blue, *E. coli* in red) (bar, 50 μm). **(D)** Confocal immunofluorescence views of the villin of the microvilli layer secreted by the Caco2 cells cultured under the conditions described in **(A)** (nuclei in blue, villin in red) (bar, 50 μm). Computerized quantification of the coverage **(E)** and height **(F)** of the sugar chain portion and villin measured under the conditions described in **(A)** (*n* = 3; **p < 0.01, ***P < 0.001). Polarized secretion of proinflammatory cytokines (IL-8, IL-6, TNF-α, and IL-1β) in the enteric cavity **(H)** and vessel lumen **(G)** under the conditions described in **(A)** (*n* = 3; *P < 0.05, **p < 0.01).

In order to investigate the effect of the probiotics on the intestinal inflammatory response, *L. casei* L5 BGB and *E. coli* were added together into the human gut-vessel microsystem. Immunofluorescence analysis on the glycans and villin suggested that the presence of *L. casei* L5 BGB significantly alleviated the damage of glycocalyx and microvilli caused by *E. coli*, and reduced the amount of *E. coli* adhering to intestinal epithelial cells after 24 h co-culture (Figures 8C–F). *L. casei* L5 BGB also enhanced intestinal barrier function, causing increased level of occludin and more than 30% reduction in the *P*_app_ value of the intestinal epithelium compared to the group without addition of *L. casei* L5 BGB (Figures 8A,B). As shown in Supplementary Figures S4A,B and Figures 8G,H, co-culture of *L. casei* L5 BGB and *E. coli*, significantly reduced (*P* < 0.001) the secretion levels of NO, TNF-α, IL-6, and IL-8 both in the intestinal lumen and vascular lumen. IL-8 was reduced by nearly 10 times in particular. Besides, a dose and a combination of antibiotics (100 units/mL penicillin and 100 μg/mL streptomycin) that effectively killed *E. coli* microbes were identified. After 24 h of cultivation in the presence of antibiotics on the microsystem, intestinal barrier function, glycocalyx and microvilli were largely protected from damage (Figures 8A–F), and there was a significant (*P* < 0.001) reduction in the secretion of NO, TNF-α, IL-1β, IL-6, and IL-8 in both the intestinal and vascular lumen (Supplementary Figures S5C,D and Figures 8G,H).

## Discussion

To establish a stable ecosystem containing physiologically differentiated intestinal epithelium, gut microbes, and immune cells that can be cultured for days to weeks is one of the critical prerequisites for mimicking the living human intestine *in vitro* (Bein et al., 2018). Here we designed and fabricated a new peristaltic human gut-vessel microfluidic device to mimic key features of human intestine for stable long-term co-culture of gut microbiome with intestinal epithelial cells. We first proposed and constructed a new peristalsis intestinal model based on principle of cyclically changing fluid pressure difference. Compared with previous models (Kim et al., 2012, 2016; Shah et al., 2016), our model is much closer to physiological conditions and simpler. We achieved fluid flow and peristalsis of the intestinal microsystem simultaneously by using a multi-channel, computer-controlled pneumatica pump. Moreover, the influences of vascular endothelial cell on differentiation of intestinal epithelial cells and development of human enteritis were studied for the first time on this microsystem.

The effects of peristalsis plus fluid flow on the growth and differentiation of intestinal epithelial cells on this device were investigated. As a traditional intestinal absorption model *in vitro*, Caco-2 cells are generally grown for at least 3 weeks in the Transwell system to differentiate into intestinal absorption functions (Hubatsch et al., 2007; Kasper et al., 2016). In clear contrast, our results demonstrated that periodic peristalsis plus fluid flow in the microfluidic device significantly promoted the proliferation of the intestinal epithelial cells and the secretion of glycocalyx and microvilli. Besides, the differentiation of the barrier, absorption and metabolism functions of intestinal epithelial cells were also influenced by periodic peristalsis plus fluid flow on the chip. This might be because the mechanically active microenvironment of living intestine (peristaltic motions and intralumenal fluid flow) might be critical for normal organ physiology (Kim et al., 2016; Wang et al., 2017). Moreover, the presence of continuous fluid flow and mechanical stretching enhanced intestinal differentiation (Huh et al., 2013) and permitted bacterial populations to reach a dynamic steady-state for a week (Kim et al., 2012).

Using this gut-vessel microsystem, it was found that the presence of vascular endothelial cell (HUVECs) improved the aminopeptidase activity of the intestinal epithelial cells (Caco-2), and also promoted the proliferation and absorption of Caco-2 cells. There are two possible reasons for these results. First, the main function of blood flow of microvessels in the body is to transmit nutrients and signals (Chrobak et al., 2006; Gray and Stroka, 2017), which are crucial for the growth and differentiation of tissue cells. Second, vascular endothelial cells and intestinal epithelium interacted with each other through the gap flow on the device, which promoted cells growth, metastasis and drug delivery (Al-Mehdi et al., 2000; Maoz et al., 2018).

Interestingly, after Caco2 cells were co-cultured with HUVECs in the microfluidic chip with peristalsis for 4 days, it was found that the originally planar columnar epithelium spontaneously grew to form undulating crypt. In contrast, spontaneous formation of intestinal glycocalyx and microvilli by Caco-2 cells was not seen in the Transwell chamber. Previous observations showed that much like in living intestine, proliferative Caco-2 cells became spatially restricted to the basal crypt niche in the gut-on-a-chip, where they generated daughter cells that migrated upward to populate the villi formed *in vitro* in the device (van der Flier and Clevers, 2009; Sato et al., 2011). On the other hand, the Caco-2 intestinal epithelial cells retain stem cell-like function, so they could faithfully recreate intestinal villi that exhibit small intestinal villus morphology and functions observed *in vivo* (Toner and Carr, 1969; Jung et al., 2011).

By introducing immune cells and *E. coli* to the gut microsystem, it was observed that the excessive proliferation of *E. coli* caused disruption of intestinal barrier, injury of intestine villus and production of a key set of proinflammatory cytokines (IL-8, IL-6, IL-1β, and TNF-α) on the microfluidic device, which were consistent with the *in vivo* and *in vitro* results reported previously (Pahl, 1999; Berkes et al., 2003; Kim et al., 2016). Interestingly, co-culture of vascular endothelial cells with intestinal epithelial cells caused a stronger inflammatory response, but both damage of the glycocalyx, microvilli and intestinal barrier were alleviated. In fact, vascular endothelial cells are known to undergo activation in response to LPS stimulation, with enhanced expression levels of cell adhesion molecules (CAM) and cytokines/chemokines (Binion et al., 1997; Ogawa et al., 2003). Moreover, as the second line of defense system, human intestinal microvascular endothelial cells can act as a mechanical barrier against pathogenic invasion, which play vital roles in both progenitor and adaptive immune defense (Heidemann et al., 2006; Maynard et al., 2012).

On this microsystem, it was found that *L. casei* as a probiotic not only could co-culture with Caco2 cells for days to a week, but also promote the secretion of intestinal glycocalyx. The results are consistent with the fact that commensal bacteria like *L. casei* can produce lactic acid and other metabolites to maintain balance in the intestinal flora (Servin, 2004), modulate mucosal (Reiff et al., 2009), promote digestion and absorption of intestinal epithelial cells (Jijon et al., 2004). Our experiments further demonstrated that *L. casei* L5 BGB could protect against overgrowth of *E. coli* and consequent gut injury, which were presumably due to the observation that *L. casei* can strengthen the gut barrier function and influence both the induction and effectors’ functions of the mucosal immune system (Gionchetti et al., 2003; Kleerebezem and Vaughan, 2009), and the supernatant of *L. casei* may exert immunological activities and strong inhibitory effect on adhesion of virulent *E. coli* strains to epithelial cell (Ingrassia et al., 2005). Our results also showed that administration of a mixture of bactericidal antibiotics suppressed inflammatory responses and intestinal injury induced by *E. coli*, demonstrating that this form of adjuvant therapy can be studied *in vitro* using the gut-on-a-chip. Taken together, these findings suggested that this human gut microsystem would be potentially useful for evaluating and screening probiotics and drugs in cure of enteritis.

## Conclusion

In summary, a novel peristaltic human gut-vessel microsystem was designed, established and put into application. By using only a pneumatic pump, peristalsis and fluid flow were achieved easily and conveniently in our device. Periodic peristalsis like *in vivo* effectively promoted the growth of intestinal epithelial cells, the differentiation of intestinal absorption function and secretion of the glycocalyx and microvilli, and the presence of endothelial cells further enhanced these responses. With introducing immune cells into the system, and *E. coli* caused disruption of intestinal barrier, injury of intestine villus and inflammatory reactions were observed on the chip microsystem. Moreover, *Lactobacillus casei* and antibiotics effectively reduced intestinal epithelial damage and inflammatory responses. Given that this peristaltic human gut-vessel microsystem can effectively recapitulate many complex functions of the normal human intestine, it would become an essential platform for studying host-microbial interaction, and development of personal medicine in the future.

## Data Availability Statement

All datasets generated for this study are included in the article/Supplementary Material.

## Author Contributions

BJ, XZ, JL, and YD designed the study. BJ, CZ, and QD performed experiments and analyzed data. ZW, JW, and YL provided an experimental resource. BJ, ZW, XZ, JL, and YD wrote and revised the manuscript.

## Conflict of Interest

The authors declare that the research was conducted in the absence of any commercial or financial relationships that could be construed as a potential conflict of interest.
