# Supplementary material for: Establishment and Application of Peristaltic Human Gut-Vessel Microsystem for Studying Host–Microbial Interaction
Source: Front Bioeng Biotechnol. 2020 Mar 31;8:272. doi: 10.3389/fbioe.2020.00272 (PMC7137556; doi:10.3389/fbioe.2020.00272)
Supplement: Supplementary file 1 [file Data_Sheet_1.PDF]

Establishment and application of peristaltic human gut-vessel microsystem for studying host–microbial interaction

Bolin Jing[1,2], Zhuo A Wang[1], Chen Zhang[1], Quanfeng Deng[3], Jinhua Wei[1], Yong Luo[3], Xiuli Zhang[4*], Jianjun Li[1*] and Yuguang Du[1*]

[1]State Key Laboratory of Biochemical Engineering, Institute of Process Engineering, Chinese Academy of Sciences, Beijing 100190, P.R. China

[2]Department of Chemistry, University of Chinese Academy of Sciences, Beijing 100049, P.R. China

[3]Key Laboratory of Fine Chemicals, Department of Chemical Engineering, Dalian University of Technology, Dalian 116024, P.R. China

[4]College of Pharmaceutical Sciences, Soochow University, Soochow, P.R. China

*** Correspondence:**
Jianjun Li, Xiuli Zhang and Yuguang Du
jjli@ipe.ac.cn (J.L.); zhangxiuli@dicp.ac.cn (X.Z.); ygdu@ipe.ac.cn (Y.D.).

Supplementary Material

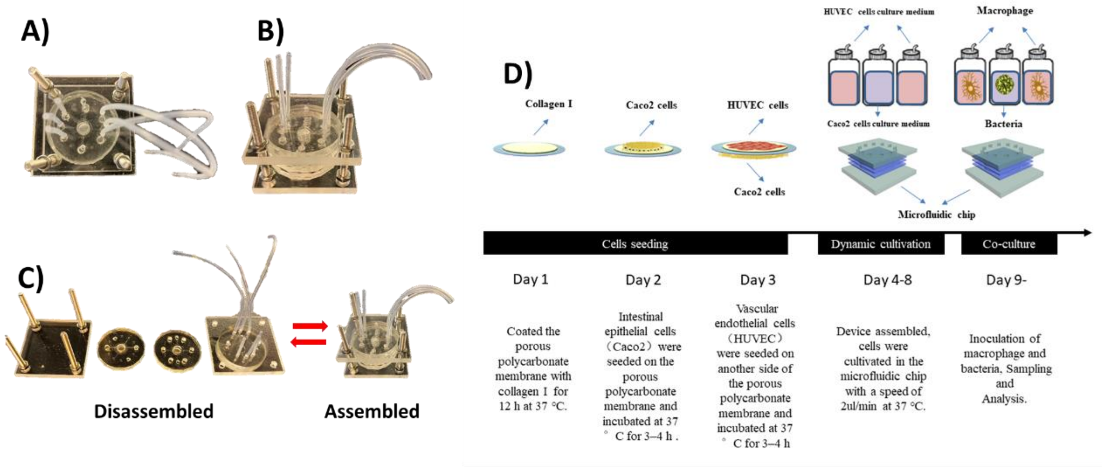

Supplementary Figure 1. (A) Top view of the actual device. (B) Side view of the actual device. (C) Physical pictures of the device disassembled and assembled with screws. (D) Diagrammatic overview of the peristaltic human gut-vessel microsystem protocol.

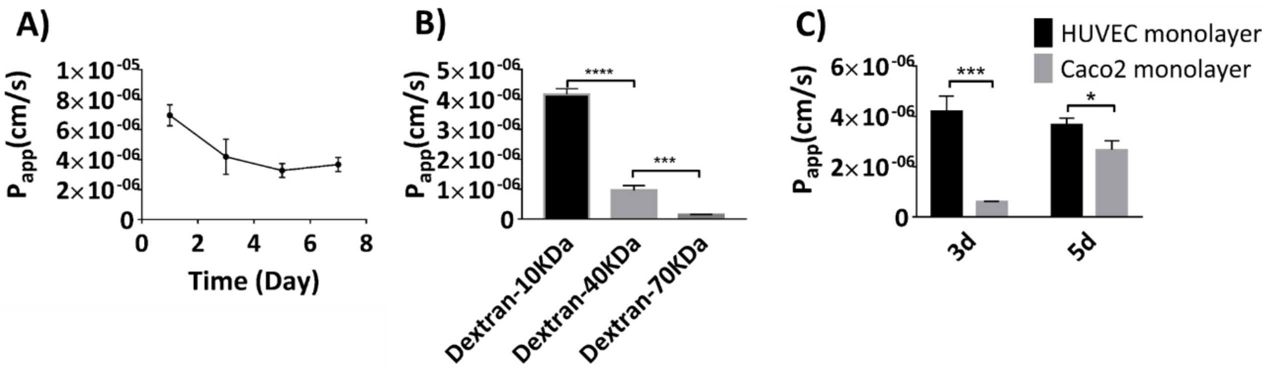

Supplementary Figure 2. (A) Apparent paracellular permeability (*Papp*) measured by real-time quantitating fluorescent dextran transport through the HUVECs monolayer cultured in the peristaltic microfluidic chip for 7 days. (B) The P_{app} values of FITC-dextrans with different molecular weight (10 kDa, 40 kDa and 70 kDa) across the endothelial monolayer on the third day (n =3; ***P < 0.001, ****P < 0.0001). (C) Quantification of *Papp* measured by quantitating fluorescent dextran (10 kDa) transport across intestinal epithelial layer or endothelial monolayer in the peristaltic microfluidic chip for 3 days and 5 days (n =3; *P < 0.05, ***P < 0.001).

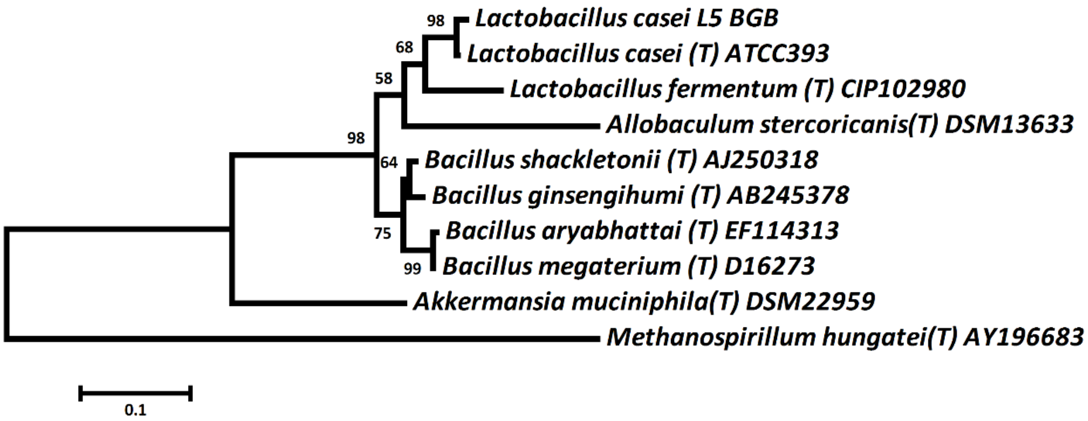

Supplementary Figure 3. Phylogenetic tree of (bacteria) the *Lactobacillus casei* L5 BGB based on 16s rRNA sequences.

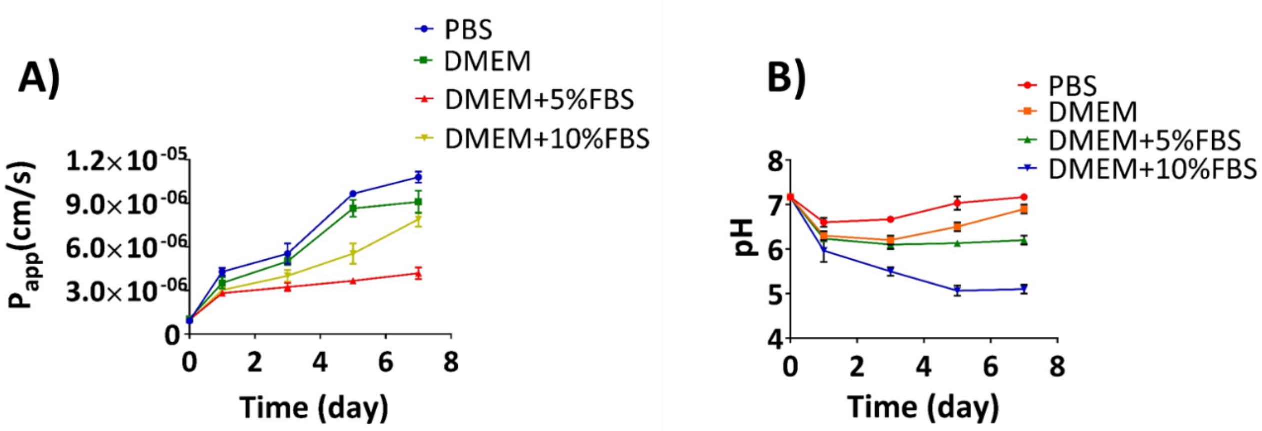

Supplementary Figure 4. (A) Changes in *Papp* measured by quantitating fluorescent dextran transport across the tissue-tissue interface within the peristaltic human gut-vessel microsystem with *L. casei* L5 BGB under different culture conditions for 7 days. (B) Changes in pH of the effluent within the peristaltic human gut-vessel microsystem with *L. casei* L5 BGB under different culture conditions for 7 days.

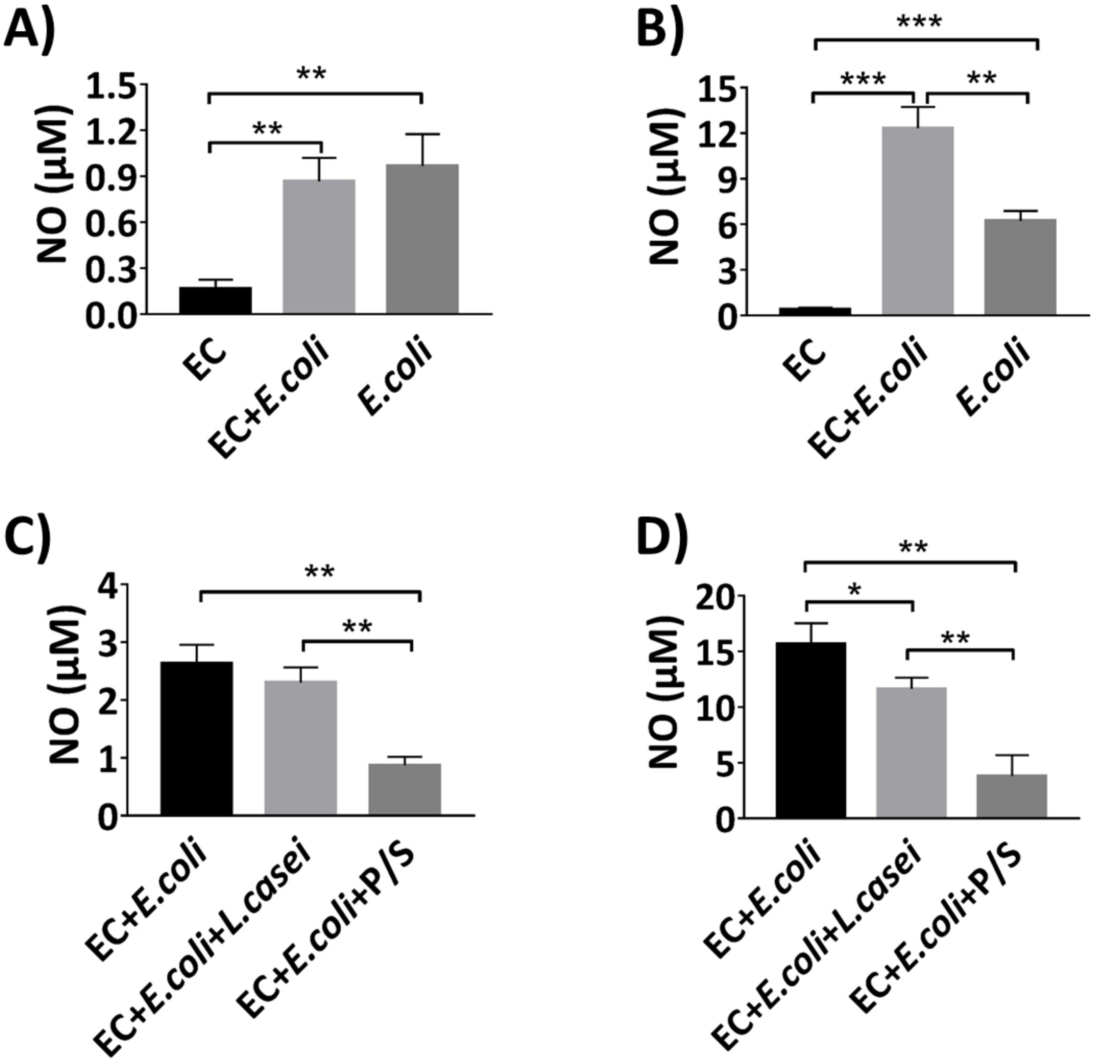

Supplementary Figure 5. Secretion of NO in the enteric cavity (A) and vascular lumen (B) in the peristaltic human gut-vessel microsystem under the control conditions (EC) versus presence of *E.coli* with (EC+ *E.coli*) or without (*E.coli*) endothelial cell for 24 hr (n = 3; **p < 0.01, ***P < 0.001). Secretion of NO in the enteric cavity (C) and vascular lumen (D) in the peristaltic human gut-vessel microsystem with *E. coli* under control conditions (EC + *E. coli*) versus presence of *L. casei* (EC + *E. coli* + *L. casei*) or P/S (EC + *E. coli* + P/S) for 24 hr (n = 3; *P < 0.05, **p < 0.01).
